# Route-Dependent Effects of Pre-Existing Anti-Ad5 Immunity on Systemic, Mucosal and Lymph Node B Cell Responses to an Ad5-Vectored Vaccine

**DOI:** 10.3390/vaccines14100893

**Published:** 2026-10-12

**Authors:** Wanru Zheng, Jingjia Xiang, Xinyang Guo, Lin Zhu, Zhe Zhang, Lihua Hou

**Affiliations:** 1 Academy of Military Medical Sciences, Beijing 100071, China; 2 College of Pharmacy, Nanjing University of Chinese Medicine, Nanjing 210023, China; 3 School of Medicine, Zhejiang University, Hangzhou 310058, China

**Keywords:** pre-existing anti-Ad5 immunity, respiratory immunization, humoral immunity, mucosal immune responses, germinal center B cell responses, memory B cell responses

## Abstract

**Background/Objectives**: Pre-existing anti-Ad5 immunity (PEI) impairs the immunogenicity of intramuscular (IM) Ad5-vectored vaccines. Whether respiratory immunization can better preserve vaccine-induced immunity remains unclear. **Methods**: We established a murine PEI model to compare immune responses following IM, intranasal (IN), or intratracheal (IT) vaccination with an Ad5-vectored SARS-CoV-2 vaccine. **Results**: PEI significantly suppressed serum receptor-binding domain (RBD)-specific IgG and pseudovirus-neutralizing antibody (pNAb) responses and germinal center (GC) B cell responses after IM vaccination. Conversely, systemic antibody responses induced by respiratory immunization were relatively less impaired, with IN vaccination accelerating seroconversion and enhancing early GC B cell activity. Unlike IM vaccination, respiratory immunization induced detectable bronchoalveolar lavage fluid (BALF) RBD-specific IgA and angiotensin-converting enzyme 2 (ACE2)-blocking activity. PEI-associated reductions in BALF IgA and ACE2-blocking activity occurred earlier after IN vaccination but later after IT vaccination. RBD-specific GC B cell responses differed across respiratory immunization, with an early enhancement followed by a late reduction after IN vaccination and a transient reduction around the apparent response peak after IT vaccination. RBD-specific memory B cells (MBCs) were broadly distributed across multiple lymph nodes. PEI-associated differences were limited to specific lymph-node sites and varied according to vaccination routes and time point. **Conclusions**: PEI suppressed systemic antibody responses after IM but less so after respiratory immunization; IN vaccination enhanced early antibody and RBD-specific GC B cell responses. Respiratory immunization induced mucosal immune responses with distinct kinetics and PEI-associated effects. These findings suggest that respiratory immunization partially mitigates the impact of PEI and provide insights that may inform the selection and optimization of vaccination routes for Ad5-vectored vaccines.

## 1. Introduction

Respiratory infectious diseases, including COVID-19, influenza, and tuberculosis, continue to impose a substantial global disease burden, causing millions of deaths each year and underscoring the urgent need for vaccines that can block pathogen transmission at the portal of entry [1]. Among the available vaccine platforms, adenovirus type 5 (Ad5) vectors have shown robust immunogenicity in clinical and preclinical studies of SARS-CoV-2, influenza, and tuberculosis, eliciting potent systemic humoral and T cell responses through highly efficient transduction [2,3,4]. However, while intramuscular (IM) vaccination can induce robust systemic immunity, it typically elicits weaker mucosal immune responses than direct respiratory immunization [5]. By contrast, mucosal immunization with Ad5 vectors induces local secretory IgA and tissue-resident memory T cells, thereby reducing viral loads in the upper airways and limiting transmission. This level of protection against respiratory viruses is not readily achieved by intramuscular administration [6,7]. Nevertheless, mucosal immune responses are not uniform throughout the respiratory tract. Intranasal (IN) and intratracheal (IT) vaccination can induce distinct immune profiles, including differences in the distribution of secretory IgA, the localization of tissue-resident memory T cells, and antibody titers in the alveolar and upper airway compartments [5,8]. Whether these route-dependent differences influence the development of systemic and mucosal immune responses remains unclear.

The clinical translation of Ad5-vectored vaccines is limited by pre-existing anti-Ad5 immunity (PEI). In many adults, high titers of anti-Ad5 neutralizing antibodies can substantially reduce vaccine-induced immune responses [9]. Pre-existing antibodies may neutralize vector particles and limit vector transduction or antigen availability. Mucosal anti-Ad5 antibodies, including secretory IgA and IgG, may also affect the activity of Ad5 vaccines delivered to the respiratory tract. Although vector engineering, alternative delivery systems, and serotype switching have been explored to mitigate anti-vector immunity [10,11,12], a clearer understanding of how PEI influences Ad5 vaccine responses remains necessary. In particular, the effects of PEI on systemic and mucosal immunity may vary with the route of vaccination, and it remains unclear whether mucosal immunization can preserve selected vaccine-induced responses. Direct comparisons in the presence of comparable systemic and mucosal anti-Ad5 antibody levels are therefore needed to define these route-dependent effects and support the optimization of Ad5-based vaccination strategies.

To address these questions, we established a murine model with comparable baseline systemic and mucosal anti-Ad5 antibody levels and systematically compared IM, IN, and IT administration of an Ad5-vectored SARS-CoV-2 vaccine. Our findings reveal that while PEI suppressed systemic humoral immunity following IM vaccination, both IN and IT immunization largely preserved these responses; notably, IN vaccination accelerated seroconversion and enhanced early germinal center B cell activity. Furthermore, unlike IM vaccination, respiratory immunization effectively elicited robust mucosal immune responses, as evidenced by detectable bronchoalveolar lavage fluid (BALF) IgA and ACE2-blocking activity, although PEI was associated with reduced mucosal responses at later time points. We also characterized route-specific temporal patterns in RBD-specific germinal center (GC) B cell responses and the distribution of memory B cells (MBCs) across various lymph nodes, noting localized PEI-associated differences. Collectively, these data suggest that respiratory immunization can mitigate the suppressive effects of PEI on Ad5-vectored vaccine-induced systemic immunity and effectively elicit mucosal immune responses. These findings provide crucial insights into the interplay between pre-existing vector immunity and vaccination routes, highlighting the potential of respiratory immunization as a promising strategy for optimizing Ad5-vectored vaccine platforms in populations with prevalent Ad5 exposure.

## 2. Materials and Methods

### 2.1. Animal Experiments

Female BALB/c mice, 6 weeks of age, were obtained from SPF Biotechnology Co., Ltd. (Beijing, China). The animals were acclimatized for 1 week and maintained under specific-pathogen-free conditions on a 12 h light–dark cycle at a constant temperature. All experimental procedures were approved by the Animal Care and Use Committee of the Academy of Military Medical Sciences.

### 2.2. Pre-Existing Immunity Model

To establish pre-existing anti-Ad5 immunity, mice received a single intranasal dose of 1 × 10^7^ ifu of an Ad5 vector in 20 μL. PEI-naïve control mice received an equivalent volume of PBS by the same route. Thirty days later, serum anti-Ad5 hexon-specific IgG titers were measured to confirm the establishment of anti-Ad5 antibody responses. Mice were then assigned to the IM, IN, or IT vaccination groups. Serum anti-Ad5 antibody levels did not differ significantly among the route-assigned PEI groups before vaccination.

### 2.3. Vaccine and Immunization

Ad5-nCoV is a human adenovirus type 5-vectored vaccine encoding the spike protein of the prototype SARS-CoV-2 strain. The vaccine was administered at a dose of 1 × 10^8^ vp per mouse by one of three routes: IM, IN, or IT. For IM immunization, 100 μL was injected into the lateral hind-thigh muscle. For IN immunization, mice were anesthetized with isoflurane, and 20 μL of vaccine was instilled dropwise into the nares, primarily targeting the upper respiratory tract. For IT immunization, mice were anesthetized with sodium pentobarbital and placed in the supine position on an inclined board. The tracheal opening was visualized using a laryngoscope (YUYAN Instrument, Shanghai, China), and 50 μL of vaccine was aerosolized using a nebulizer (YUYAN Instrument) positioned 25 mm beyond the larynx, near the carina, to directly target the lungs. The vaccine dose was matched across the three administration routes, whereas the administration volume and procedure were route-specific.

### 2.4. Sample Collection and Processing

At each time point (days 7, 14, 28, and 56), 10 mice per group were sacrificed, with 5 mice sacrificed on day 0, unless otherwise noted. Serum, BALF, and lymph nodes were collected from the same mouse. Blood samples were collected first from the tail vein, after which the mice were euthanized by cervical dislocation, followed by collection of BALF and lymph node samples. Serum was separated from the collected blood by centrifugation at 1500× *g* for 10 min at 4 °C. For BALF collection, the trachea was exposed and lavaged twice with 200 μL PBS, and the BALF samples were separated by centrifugation at 500× *g* for 5 min at 4 °C. For RBD-specific GC B cell analysis, the primary draining lymph nodes were harvested at all time points, consisting of the iliac lymph node for the IM group, cranial deep cervical lymph node for the IN group, and mediastinal lymph node for the IT group. For RBD-specific MBCs analysis on days 28 and 56, the mandibular, deep cervical, inguinal, iliac, popliteal, and mediastinal lymph nodes were harvested.

### 2.5. Enzyme-Linked Immunosorbent Assay (ELISA)

Ad5 hexon-specific IgG and SARS-CoV-2 RBD-specific IgG and IgA were quantified by ELISA. Briefly, plates were coated overnight at 4 °C with 1 μg/mL recombinant Ad5 hexon or RBD and then blocked with 2% BSA in PBST for 1 h at 37 °C. Serially diluted samples were incubated for 1 h at 37 °C, followed by incubation with HRP-conjugated goat anti-mouse IgG or IgA (1:10,000; Abcam, Cambridge, UK) for 1 h at 37 °C. Signals were developed with TMB substrate for 6–8 min at 37 °C; the reaction was stopped, and absorbance was measured at 450/630 nm using a Molecular Devices reader. Endpoint titers were defined as the highest reciprocal dilution yielding an OD at least 2.1-fold higher than the mean OD of pre-immune serum. For negative samples, a value corresponding to one-half of the starting dilution was assigned.

### 2.6. SARS-CoV-2 Pseudovirus Neutralization Assay

Heat-inactivated serum (56 °C, 30 min) was serially diluted 3-fold starting at 1:30 and mixed with an equal volume of 100 TCID_50_ SARS-CoV-2 pseudovirus (Vazyme, #DD1702, Nanjing, China). After incubation for 1 h at 37 °C, HEK293T/ACE2 cells were added and cultured for 48 h. Luciferase activity was measured using the Bright-Lite Assay System (Vazyme, #DD1204) on a Promega luminometer. EC_50_ titers were calculated by the Reed–Muench method. Seronegative samples were assigned half the initial dilution.

### 2.7. ACE2 Receptor-Binding Inhibition Assay

BALF samples were analyzed using a commercial ACE2 receptor-binding inhibition kit (Vazyme, #DD3101) according to the manufacturer’s instructions. Diluted samples were pre-incubated with HRP-conjugated RBD for 30 min at 37 °C, transferred to hACE2-coated plates, and incubated for another 30 min at 37 °C. After washing, TMB substrate was added and incubated for 15 min at 37 °C before the reaction was stopped. Absorbance was measured at 450 nm using a Molecular Devices reader. The percentage inhibition relative to the negative control was calculated. Samples with inhibition of at least 20% were considered positive, while those below this threshold were classified as negative.

### 2.8. Flow Cytometric Identification of RBD-Specific GC B Cells and MBCs

RBD-specific B cells were identified using biotinylated RBD multimerized with streptavidin-fluorophore conjugates (2:1 mass ratio). Excess streptavidin was quenched with 5 μM free D-biotin (Avidity LLC, #BIRA500, San Diego, CA, USA) after 1 h at 4 °C. Single-cell suspensions from multiple lymph nodes were prepared by mechanical disaggregation and 70 μm filtration. Cells were first blocked with anti-CD16/32 (BD Biosciences, #553142, San Jose, CA, USA) and stained with NIR viability dye (Thermo Fisher, #L34976, Waltham, MA, USA) for 30 min on ice, then probed with 25 ng each of RBD-Alexa Fluor 647 and RBD-FITC for 1 h at 4 °C. Surface staining included anti-CD4 BV510 (1:400; RM4-5, BioLegend, San Diego, CA, USA), anti-CD19 BV605 (1:100; 6D5, BioLegend), anti-Fas PE (1:100; SA367H8, BioLegend), anti-GL7 PerCP-Cy5.5 (1:100; GL7, BioLegend), anti-IgD BV421 (1:800; 11-26c.2a, BioLegend), anti-CD38 Alexa Fluor 700 (1:400; 90, BioLegend), and anti-CD138 PE-Cy7 (1:400; 281-2, BioLegend) for 30 min at 4 °C. Data were acquired on a BD FACSCanto II and analyzed with FlowJo v10.8.1. RBD-specific GCBs and MBCs were defined as live CD4-CD19 + GL7 + Fas + RBD + and IgD-CD138-CD19 + GL7-CD38 + RBD + cells, respectively.

### 2.9. Statistical Analysis

Statistical analyses were performed using GraphPad Prism 10.5.0. Antibody titers were log-transformed before analysis. Percentage inhibition values were analyzed without transformation. Group differences involving PEI status, vaccination routes, lymph node station, and/or time point were evaluated using two-way ANOVA followed by Sidak’s multiple-comparisons test, or the unpaired Mann–Whitney U test for direct comparisons between PEI and PEI-naïve groups, as appropriate. Correlations were assessed using Pearson’s or Spearman’s correlation analysis, as indicated in the figure legends. Seropositivity rates were summarized descriptively. Samples below the assay detection limit were assigned the prespecified values described in the corresponding assay methods. Statistical significance was defined as *p* < 0.05.

## 3. Results

### 3.1. Establishment of a Mouse Model of Pre-Existing Anti-Ad5 Immunity

To evaluate the impact of pre-existing Ad5 immunity (PEI) on vaccine-induced responses, mice were primed intranasally with an Ad5 vector or PBS as a control. One month later, PEI and PEI-naïve mice were immunized with an Ad5-based SARS-CoV-2 vaccine at a dose of 1 × 10^8^ viral particles (vp) per mouse via the IM, IN, or IT route. IN and IT administration were compared as distinct respiratory immunization approaches, with IN immunization initiated through the nares and primarily targeting the upper respiratory tract, whereas IT immunization was performed through the trachea to directly target the lungs. Serum, BALF, and multiple draining lymph nodes were collected on days 0, 7, 14, 28, and 56 after vaccination (Figure 1a). Serum anti-Ad5 antibody titers measured 30 days after Ad5 priming and immediately before vaccination were comparable among all PEI-positive subgroups assigned to IM, IN, or IT vaccination, as well as among the prespecified sacrifice time points (Figure 1b). These data confirmed the establishment of PEI and comparable baseline serum anti-Ad5 antibody levels before vaccination. In addition, analysis of 20 randomly selected PEI mice showed strong positive correlations between serum Ad5 IgG and both BALF Ad5 IgG (r = 0.8596, *p* < 0.0001) and BALF Ad5 IgA (r = 0.8493, *p* < 0.0001) (Figure 1c), indicating that systemic PEI was associated with mucosal anti-Ad5 antibody levels in this subset of PEI mice. Together, these data established a model with comparable serum anti-Ad5 antibody levels before vaccination and demonstrated an association between systemic and mucosal anti-Ad5 antibody responses.

### 3.2. PEI Suppresses Systemic Antibody Responses After Intramuscular but Not Respiratory Immunization

Having established the PEI model, we next examined the effects of PEI on systemic antibody responses after vaccination. For serum RBD-specific IgG, the IM PEI-naïve group had a geometric mean titer (GMT) above 10^3^, whereas the IN and IT PEI-naïve groups had lower GMTs at day 7 (Figure 2a). A significant PEI status and vaccination routes interaction emerged at day 28 (*p* = 0.0319), indicating that the effect of PEI differed among the three vaccination routes at this time point. This interaction was mainly characterized by a marked reduction in IgG responses in the IM PEI group, with the GMT in the corresponding IM PEI-naïve group being 4.5-fold higher (*p* = 0.0007). By day 56, the IgG GMT in the IM PEI-naïve group remained approximately 3.4-fold higher than that in the corresponding IM PEI group. No significant PEI-associated differences were observed in the IN or IT groups through day 7 to day 56. Thus, the route-dependent effect of PEI on serum RBD-specific IgG was most evident on day 28, while the suppressive pattern in the IM group persisted through day 56. Compared with serum RBD-specific IgG responses, pseudovirus-neutralizing antibody (pNAb) responses showed a more pronounced temporal PEI status and vaccination routes interaction, with significant interactions at days 7 (*p* = 0.0082), 14 (*p* = 0.0258), and 28 (*p* = 0.0273), respectively (Figure 2b). In the IM group, the PEI-naïve group had higher pNAb GMTs at every time point, with GMTs approximately 5.2-fold higher than those in the PEI group on day 28 (*p* = 0.0008). No PEI-associated suppression was observed in either mucosal vaccination group. Notably, PEI was associated with enhanced early antibody responses after IN vaccination. The pNAb GMTs in the PEI-naïve group were only 0.2- and 0.4-fold those in the PEI group at days 7 (*p* = 0.0071) and 14, respectively. A concordant increase was observed for RBD-specific IgG, with the GMT in the PEI-naïve group being 0.3-fold that in the PEI group at day 7.

The pNAb-to-RBD-specific IgG ratio, used as a descriptive measure of neutralizing activity relative to RBD-binding antibody levels, showed distinct patterns across vaccination routes and PEI groups (Figure 2c). In PEI-naïve mice, the IM group showed a sustained increase in this ratio from day 7 to day 56. By contrast, the IN and IT groups showed little change between days 7 and 14, suggesting a different temporal pattern in the relative neutralizing activity of antibodies induced by mucosal and IM vaccination. PEI was associated with persistently lower pNAb-to-RBD-IgG ratios in the IM group at all measured time points, indicating lower relative neutralizing activity among RBD-binding antibodies. The corresponding ratios in the IN and IT groups did not differ between PEI and PEI-naïve mice. Notably, the IN PEI group showed early kinetics distinct from those of the IM group, with higher pNAb-to-RBD-IgG ratios than the IN PEI-naïve group on days 7 and 14. Serum pNAb seroconversion rates were consistent with these differences in pNAb response (Figure 2d). All PEI-naïve mice had seroconverted by day 28. In PEI mice, the IN group showed the most rapid seroconversion, with 90% of mice positive by day 7, compared with 40% in the corresponding PEI-naïve group, and 100% positivity by day 14. The IT PEI group reached complete seroconversion by day 28, whereas seroconversion was delayed and incomplete in the IM PEI group, with detectable pNAb activity observed in only 78% of mice at day 56. Together, PEI was associated with reduced systemic antibody responses after IM vaccination, whereas no comparable reduction was observed after IN or IT vaccination.

### 3.3. PEI Differentially Shapes the Kinetics of Mucosal IgA and ACE2-Blocking Activity After in Versus IT Vaccination

Given that systemic antibody titers do not necessarily reflect mucosal antibody responses, we assessed local humoral responses in BALF. Both mucosal vaccination routes induced detectable BALF IgG responses by day 14 and maintained numerically higher titers than those observed after IM vaccination through day 56 (Figure 3a). Although PEI significantly reduced serum IgG responses in the IM group, it did not significantly alter BALF IgG levels after IM vaccination. Consistent with the higher serum IgG response observed in IN PEI mice, PEI was associated with increased BALF IgG after intranasal immunization at days 7 and 14. Unlike BALF IgG, BALF IgA showed a significant interaction between PEI status and vaccination routes on day 28 (*p* = 0.0171) (Figure 3b). BALF IgA was undetectable after IM vaccination but was induced by both mucosal routes. The IgA response was delayed after IT vaccination, whereas comparable levels were observed in PEI-naïve mice receiving IN or IT vaccination from days 28 to 56. ACE2-blocking activity broadly followed the kinetics of the IgA response (Figure 3c). However, the IT group showed higher mean activity than the IN group from days 28 to 56 despite similar IgA levels, suggesting that total BALF IgA did not fully explain the observed ACE2-blocking activity. PEI was associated with lower BALF IgA levels and ACE2-blocking activity in the IN group at day 28 (*p* = 0.0152), whereas the corresponding reductions in the IT group occurred later, at day 56.

We next examined the correlations between ACE2-blocking activity and BALF IgG or IgA levels. When PEI and PEI-naïve mice were analyzed together, both BALF IgG and IgA levels were positively correlated with ACE2-blocking activity in the IN and IT groups. The correlation between BALF IgA and ACE2-blocking activity was stronger in the IT group (r = 0.5779, *p* < 0.0001) than in the IN group (r = 0.4031, *p* = 0.0002) (Figure 3d). To further confirm these relationships, we stratified the analyses by PEI status. In the IN group, the relationship between BALF IgG and ACE2-blocking activity was broadly similar in PEI and PEI-naïve mice (Figure 3e). By contrast, a significant association between BALF IgA and ACE2-blocking activity was observed only in PEI mice (r = 0.5217, *p* = 0.0006), in which both BALF IgA levels and ACE2-blocking activity were reduced. In the IT group, a strong association between BALF IgA and ACE2-blocking activity was evident in PEI-naïve mice (r = 0.7060, *p* < 0.0001) but was weaker in PEI mice (r = 0.4402, *p* = 0.0057). These findings suggest an association between BALF IgA responses and ACE2-blocking activity induced by mucosal vaccination, with the strength of this association varying by vaccination routes and PEI status.

### 3.4. PEI Differentially Shapes the Timing of RBD-Specific Germinal Center B Cell Responses Across Vaccination Routes

To assess the effects of PEI on vaccine-induced GC responses, we quantified the frequency of total and RBD-specific GC B cells (GCBs) in the draining lymph nodes associated with each immunization route. In the IM group, total and RBD-specific GCB frequencies in PEI-naïve mice peaked on day 14 and subsequently declined to near-baseline levels by day 56 (Figure 4a). PEI was not associated with a statistically significant difference in RBD-specific GCB frequencies on day 7 but was associated with lower RBD-specific GCB frequencies on days 14, 28, and 56 than in PEI-naïve controls (*p* = 0.0109, *p* = 0.0001, and *p* = 0.0304, respectively). After IN immunization, total GCBs in PEI-naïve mice peaked on day 14 and remained at a comparable level until day 28 (Figure 4b). PEI was associated with higher frequencies of total GCBs (*p* = 0.0001) and RBD-specific GCBs (*p* = 0.0227) on day 7, consistent with the enhanced early antibody response, whereas lower frequencies of RBD-specific GCBs were observed on day 56 (*p* = 0.0474). Thus, PEI caused an early increase followed by a late decline in RBD-specific GCB frequencies in the IN response. In the IT group, both total GCBs and RBD-specific GCBs in PEI-naïve mice reached levels similar to those observed with the other routes by day 14 and showed a sustained increase, peaking at day 28 (Figure 4c). PEI was associated with lower frequencies of total GCBs (*p* = 0.0232) and RBD-specific GCBs (*p* = 0.0011) specifically on day 28, corresponding to the apparent peak of the GC response in this group.

Analysis of the proportion of RBD-specific GCBs among total GCBs further revealed route-dependent temporal patterns in the effect of PEI (Figure 4d). In the IM group, the proportion of RBD-specific GCBs was similar between PEI and PEI-naïve mice on day 7 but was lower in PEI mice on day 14 (*p* = 0.0029). The difference reached statistical significance on day 28 (*p* = 0.0001) and persisted through day 56 (*p* = 0.0089), indicating that the proportion of antigen-specific GCBs was reduced from the mid-phase of the response onwards after IM vaccination. The mucosal routes showed different temporal profiles. In the IN group, the proportion of RBD-specific GCBs was lower in PEI mice than in PEI-naïve mice only on day 56 (*p* = 0.0316), suggesting a predominantly late reduction in antigen-specific GCB frequency. In the IT group, the proportions were similar between the two groups on days 7 and 14 but were lower in PEI mice on day 28 (*p* = 0.0232), coinciding with the apparent peak of the GC response. Together, these data indicate that PEI was associated with reductions in the frequency of RBD-specific GCBs at different phases of the GC response depending on the route of administration.

### 3.5. Distribution of RBD-Specific Memory B Cell Responses Across Lymph Nodes

Because antigen-specific memory B cells (MBCs) can be detected in multiple lymph nodes after immunization, we analyzed RBD-specific MBC responses in lymph nodes located near and distant from the administration sites. RBD-specific MBCs induced through the three immunization routes showed distinct route- and time-dependent distribution patterns. In the IM group, consistently detectable MBC responses were observed in the designated primary draining iliac lymph nodes at day 28, whereas responses in other lymph node stations were weak or variable (Figure 5a). By day 56, RBD-specific MBC responses were detectable in multiple lymph nodes, including the inguinal, popliteal, mandibular, and mediastinal lymph nodes. Thus, RBD-specific MBC responses were detected both in lymph nodes near the intramuscular injection site and in lymph nodes associated with respiratory tissues. The IN and IT groups showed weak and variable MBC responses on day 28 (Figure 5b,c). In the IN group, only the popliteal lymph nodes showed sporadic responses, with considerable inter-individual variability. By day 56, both mucosal groups showed readily detectable RBD-specific MBC responses in most of the lymph nodes examined. The IT group showed strong MBC responses in the mediastinal lymph nodes but minimal responses in the cranial deep cervical lymph nodes on day 56 (Figure 5c). In the IN group, RBD-specific MBCs were detectable in both the deep cervical and mediastinal lymph nodes at this time point, indicating distinct patterns of MBC distribution between the IN and IT groups at this time point.

The effects of PEI on RBD-specific MBC responses were limited and varied across immunization routes and time points. No clear PEI-associated alteration was detected after IM immunization at either time point. After respiratory immunization, PEI-associated reductions were confined to the popliteal lymph nodes but occurred at different stages. After IN immunization, the reduction was significant on day 28 (*p* = 0.0439) and persisted numerically on day 56 without reaching statistical significance. In contrast, after IT immunization, a significant reduction emerged on day 56 (*p* = 0.0360). MBC responses at the other lymph-node sites remained broadly comparable between PEI and PEI-naïve mice. Notably, unlike the GC B cell changes observed in the route-specific draining lymph nodes, these MBC differences were detected in the popliteal lymph nodes. Overall, RBD-specific MBCs were broadly distributed across lymph nodes, whereas PEI-associated differences remained limited to specific lymph nodes by day 56 after vaccination.

## 4. Discussion

In this study, we examined how vaccination routes influence immune responses to an Ad5-vectored SARS-CoV-2 vaccine in mice with pre-existing anti-Ad5 immunity. IM vaccination served as the systemic comparator for the two respiratory immunizations, allowing the immune responses induced by respiratory immunization to be evaluated relative to a conventional vaccination route. IN and IT vaccinations delivered the vaccine through the nares and trachea, respectively. These two respiratory routes were selected to compare immune responses following vaccine immunization to different regions of the respiratory tract, with IN primarily targeting the upper respiratory tract and IT immunization targeting the lungs. As a result, intramuscular vaccination induced more rapid systemic antibody responses in PEI-naïve mice but was more strongly affected by PEI, as reflected by systemic binding and pNAb responses. By contrast, systemic antibody responses were less affected after IN and IT vaccination. Mucosal responses, however, varied by route and time, with PEI-associated changes in BALF IgA and ACE2-blocking activity. Collectively, the effect of PEI varied with both vaccination routes and immune compartments.

PEI can reduce the immunogenicity of Ad5-vectored vaccines, although the effect varies with the vaccine construct, vector dose, administration route, and baseline antibody levels [9,13,14]. Conversely, intranasal administration has been reported to mitigate PEI-associated reductions in vaccine-induced responses [15]. Our findings are consistent with these observations and extend to IT vaccination, which also produced less-affected systemic antibody responses in PEI mice. In this study, PEI was induced intranasally, and anti-Ad5 antibodies were detectable in BALF and positively correlated with serum antibody levels, indicating that anti-Ad5 immunity was present at the mucosal surface. Despite detectable mucosal anti-Ad5 antibodies, systemic antibody responses were less affected after mucosal vaccination than those after IM vaccination. One possible explanation is that the different immunization routes resulted in distinct patterns of vector deposition and biodistribution in the respiratory tract. This possibility is consistent with a previous study showing that intranasal and intratracheal immunization of an Ad5-vectored vaccine surrogate produced different distribution profiles in the mouse lung and was associated with different local immune outcomes [16,17]. Differences in local vector deposition may influence the amount of vector available for cellular uptake, transgene expression, and antigen presentation. These data link vaccination routes to the extent of PEI-associated systemic suppression but do not define the underlying mechanism.

Although mucosal vaccination preserved systemic antibody responses in PEI mice, local antibody responses were not uniformly preserved. BALF RBD-specific IgG was not significantly reduced, whereas IgA levels and ACE2-blocking activity showed route- and time-dependent differences. This selective effect of IgA may reflect the distinct regulatory requirements of IgA class switching, which depends on local signals such as transforming growth factor-β and may be more sensitive to changes in the tissue environment than IgG responses [18]. PEI-associated changes were more pronounced in GC B cell responses than in some BALF antibody measurements. This suggests that local antibody levels may not be determined solely by GC responses in the draining lymph nodes. Pulmonary plasma cells may provide a local source of antibody during respiratory immune responses [19]. However, their precise contributions to BALF antibody levels in this model remain unclear because local antibody-secreting cells were not assessed.

After IN vaccination, PEI mice had higher serum RBD-specific IgG and neutralizing antibody titers than PEI-naïve mice between days 7 and 14. GC responses also increased earlier, with a difference on day 7. We hypothesize that this could be driven by enhanced uptake of pre-existing IgG–Ad5 complexes by Fcγ receptor-expressing antigen-presenting cells in the upper respiratory tract, which may enhance early antigen presentation and promote GC initiation [20]. No comparable early enhancement was detected after IT vaccination. This difference may reflect distinct patterns of vector deposition, uptake, and antigen presentation in the upper and lower respiratory tracts. Alveolar macrophages may also influence the uptake and clearance of inhaled vector–antibody complexes. Direct measurements of vector uptake, Fcγ receptor engagement, transgene expression, and transduction will be needed to test this hypothesis. These findings suggest that IN vaccination may be associated with enhanced early systemic antibody responses in the presence of pre-existing anti-Ad5 immunity.

MBCs are an important component of durable immunological memory [21]. Although IM vaccination induced systemic antibody responses earlier than the mucosal routes, RBD-specific MBCs did not show a similar early advantage. All three routes produced weak or variable MBC responses on day 28, whereas more robust responses were observed by day 56. This delay is consistent with the possibility that the generation and accumulation of MBCs depend on the kinetics of GC maturation rather than solely on the speed of initial activation [22]. RBD-specific MBCs were not restricted to the local draining lymph nodes but were detected at multiple sites after both IM and mucosal vaccination. This distribution is consistent with the dissemination of vaccine-induced MBCs beyond the primary draining lymph nodes and may contribute to memory across both mucosal and systemic sites [23,24]. However, the present analysis cannot distinguish active recirculation from local generation, retention, or differential survival of MBCs at the sampled sites. On day 56, PEI did not significantly reduce MBC responses in the mediastinal lymph nodes after mucosal vaccination, whereas PEI-associated differences were observed at selected lymph node sites, particularly the popliteal lymph nodes. Persistent antigen or local immune niches may contribute to the maintenance of MBC responses in the mediastinal lymph nodes. Antigen retention by follicular dendritic cells may be one factor involved in this process [25,26,27]. Together, these findings suggest that the influence of PEI on vaccine-induced MBC responses is dependent on lymph node location, with respiratory-associated lymph nodes showing relative preservation of MBC responses after mucosal vaccination.

Our study has several limitations. First, the study was performed in mice, and the findings may not fully reflect human respiratory mucosal immunity. Further evaluation in translational models, including non-human primates, is needed [28,29,30]. Second, we systematically compared the kinetics of immune responses, tissue distribution, and selected cellular subsets after mucosal and systemic vaccination. The resulting phenotypic patterns suggest potential mechanisms involving local antigen uptake and antigen retention in lymph nodes, but these mechanisms were not directly tested. Third, the study focused on an Ad5-vectored vaccine, and whether the findings extend to other viral vectors or non-viral mucosal immunization systems remains unknown. Finally, we measured humoral and cellular immune responses but did not assess protection against infection or transmission. Consequently, the actual protective value of the observed route-dependent responses requires further investigation.

## 5. Conclusions

Overall, in this murine model, PEI had route-dependent effects on vaccine-induced responses. PEI suppressed systemic antibody responses after IM but less so after IN or IT vaccination. Notably, IN vaccination induced stronger early antibody responses, accompanied by increased early RBD-specific GC B-cell responses. Respiratory immunization induced BALF IgA and ACE2-blocking activity, although their kinetics and PEI-associated changes differed. MBCs were broadly distributed across lymph nodes, with limited PEI-associated differences. These findings suggest that respiratory immunization partially mitigates the impact of PEI and provide a basis for future studies on selecting vaccination routes and designing Ad5-vectored immunization strategies.

## Figures and Tables

**Figure 1:**
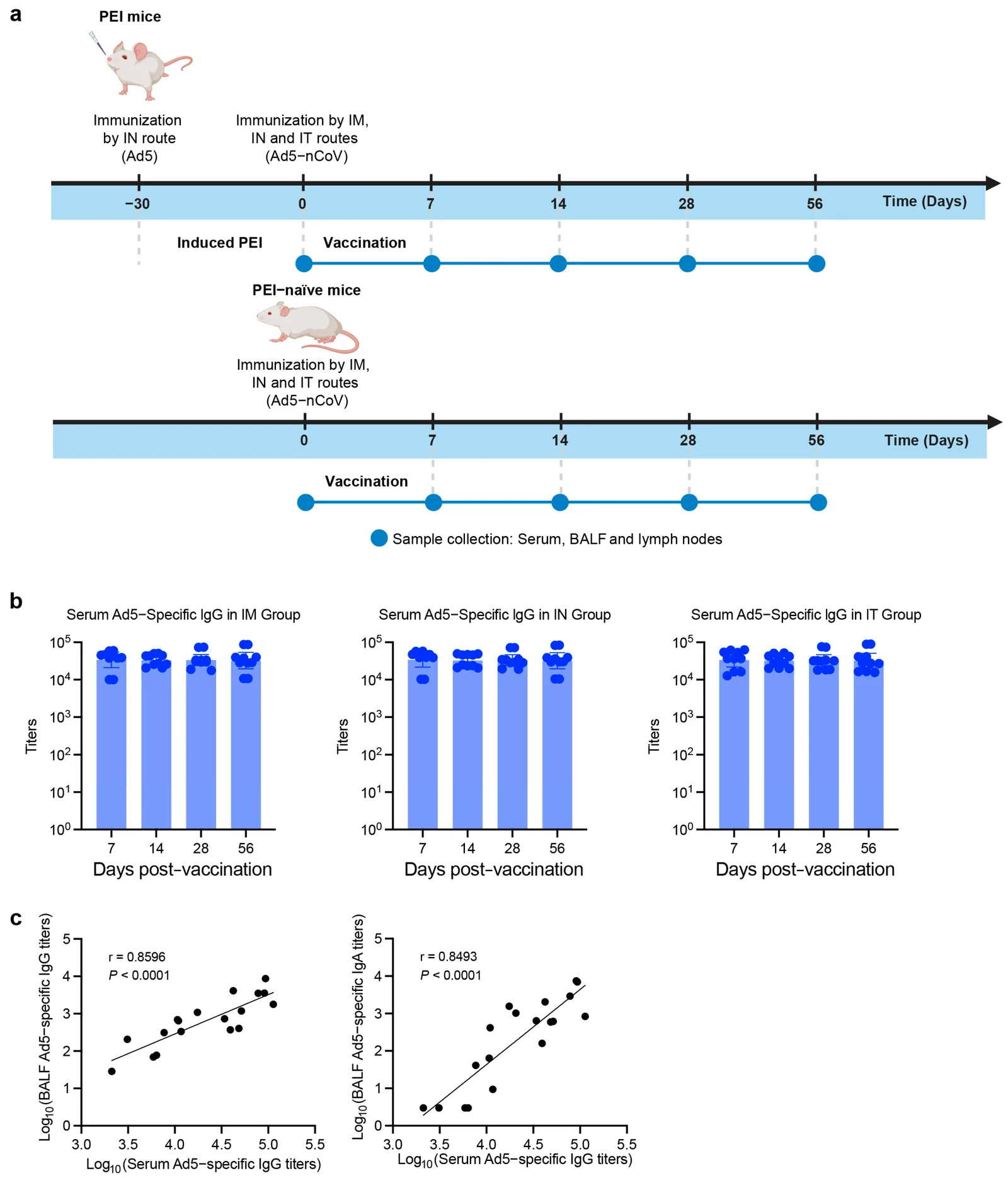
Establishment of the pre-existing anti-Ad5 immunity model and characterization of baseline antibody responses across immunization routes: (**a**) Schematic of PEI induction, vaccination, and sample collection. PEI mice received a single intranasal dose of an Ad5 vector, whereas PEI-naïve controls received an equivalent volume of PBS on day −30. This 30-day interval preceded Ad5-nCoV immunization on day 0 and allowed the pre-existing anti-Ad5 antibody response to become established before vaccination. After a 30-day interval, mice were immunized with Ad5-nCoV by the IM, IN, or IT route. Serum, BALF, and lymph nodes were collected on days 0, 7, 14, 28, and 56 after vaccination. (**b**) Serum Ad5 hexon-specific binding IgG titers measured immediately before Ad5-nCoV vaccination and 30 days after PEI induction. Individual data points represent individual mice; bars indicate GMTs with 95% confidence intervals for the IM, IN, and IT groups. Data were pooled from two independent experiments. (**c**) Pearson correlation analysis of hexon-specific binding-antibody titers in a randomly selected subset of PEI mice (*n* = 20 total). Left, correlation between serum and BALF hexon-specific IgG titers. Right, correlation between serum hexon-specific IgG and BALF hexon-specific IgA titers. Solid lines indicate linear regression fits. Statistical significance was assessed using Pearson’s correlation test.

**Figure 2:**
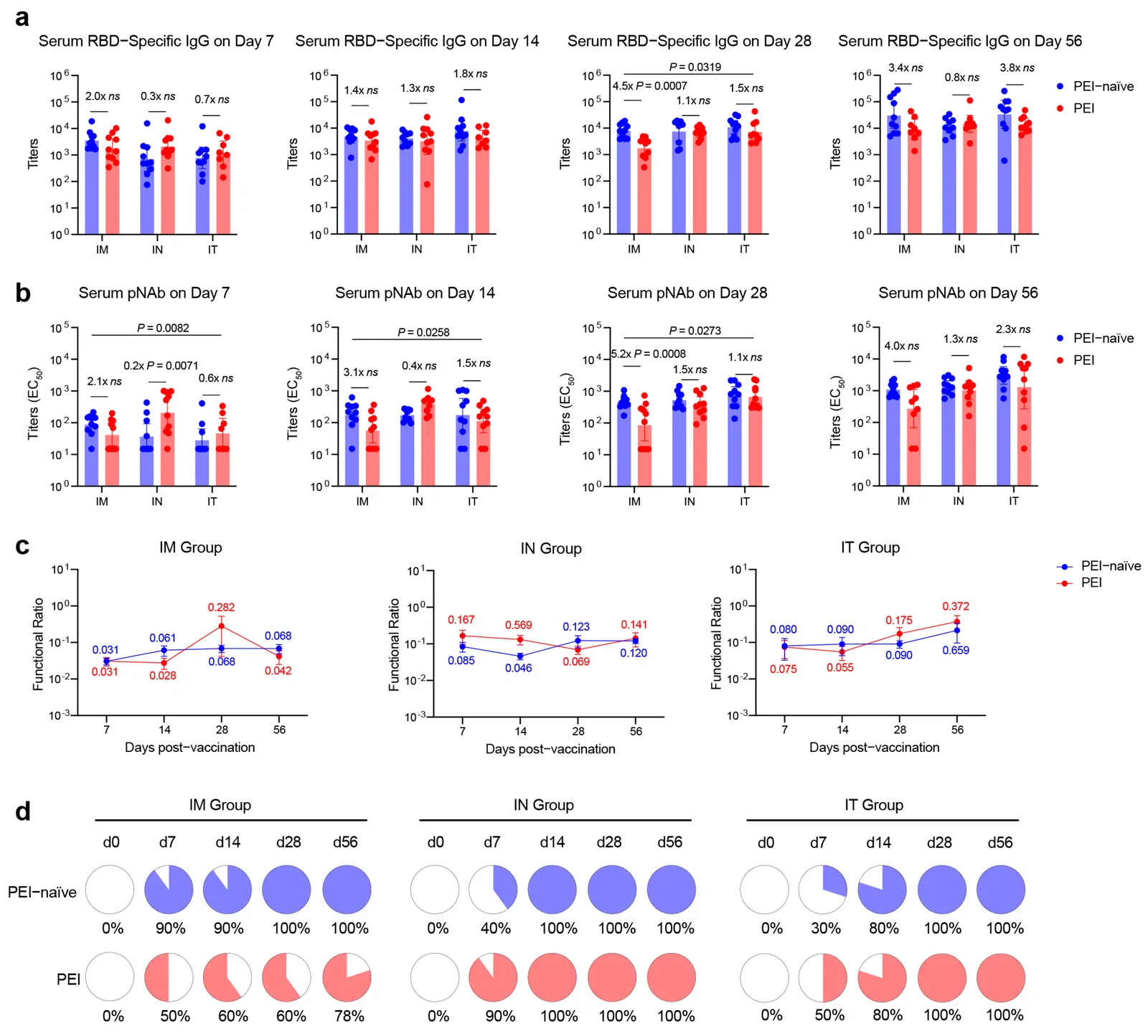
Systemic antibody responses after Ad5-nCoV immunization by three routes in PEI and PEI-naïve mice: (**a**,**b**) Serum RBD-specific IgG (**a**) and pNAb (**b**) titers in PEI (red) and PEI-naïve (blue) mice on days 7, 14, 28, and 56 after vaccination by the IM, IN, or IT route. Bars represent GMTs with 95% confidence intervals, and individual data points are overlaid. Numbers above the bars indicate the fold difference in GMTs between the PEI-naïve and PEI groups at each time point, calculated as GMT in PEI-naïve mice divided by GMT in PEI mice. Two-way ANOVA followed by Sidak’s multiple-comparison test was performed on log-transformed titers to evaluate the interaction between PEI status and vaccination routes at each time point. The significant *p* values for the interactions are displayed at the top of each panel. Exact *p* values for multiple comparisons are shown above the corresponding comparisons; ns indicates *p* ≥ 0.05. (**c**) Temporal changes in the pNAb-to-RBD-specific IgG ratio in PEI (red) and PEI-naïve (blue) mice on days 7, 14, 28, and 56 after vaccination by the IM, IN, or IT route. Points indicate group means, and error bars indicate the SEM. Values adjacent to the data points indicate the mean ratio for each group at each time point. (**d**) Serum pNAb seropositivity rates in PEI-naïve (blue) and PEI (red) mice after IM, IN, or IT immunization. Pie charts show the proportions of seropositive (colored) and seronegative (white) mice; percentages below the chart indicate seropositivity rates.

**Figure 3:**
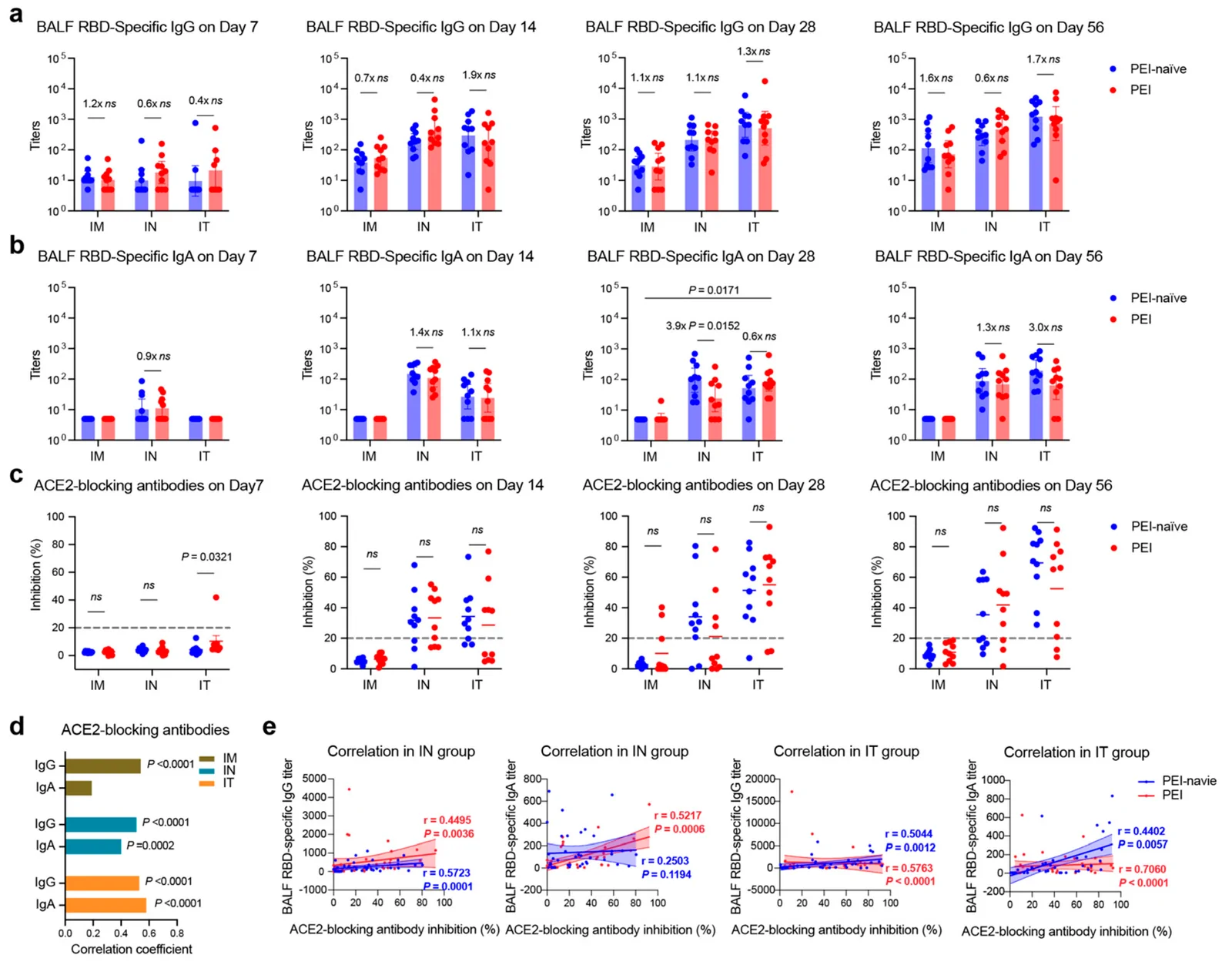
Mucosal antibody responses and ACE2-blocking activity after Ad5-nCoV immunization by three routes in PEI and PEI-naïve mice: (**a**,**b**) RBD-specific IgG (**a**) and IgA (**b**) titers in PEI (red) and PEI-naïve (blue) mice on days 7, 14, 28, and 56 after immunization by the IM, IN, or IT route. Bars indicate GMTs with 95% confidence intervals, and individual data points are overlaid. Numbers above the bars indicate the fold differences in GMTs between the PEI-naïve and PEI groups at each time point, calculated as GMT in PEI-naïve mice divided by GMT in PEI mice. (**c**) BALF ACE2-blocking activity measured using a competitive ELISA. Individual data points represent percentage inhibition values for PEI-naïve (blue) and PEI (red) mice; short horizontal lines indicate group means. The gray dashed line at 20% inhibition denotes the assay cut-off. Samples below this threshold were classified as negative or below the assay detection limit. Two-way ANOVA followed by Sidak’s multiple comparisons test was performed on log-transformed titers (**a**,**b**) or untransformed percentage inhibition values (**c**) to evaluate the interaction between PEI status and vaccination routes at each time point. The significant *p* values for the interactions are displayed at the top of each panel. Exact *p* values for multiple comparisons are shown above the corresponding comparisons; ns indicates *p* ≥ 0.05. (**d**) Spearman correlation coefficients for the associations between BALF ACE2-blocking activity and RBD-specific IgG or IgA titers across PEI and PEI-naïve mice in the IM, IN, and IT groups. Bar length indicates Spearman’s ρ; *p* values are shown to the right of each bar. (**e**) Spearman correlation analysis of BALF ACE2-blocking activity with RBD-specific IgG or IgA in IN and IT mice. Red and blue denote PEI and PEI-naïve groups, respectively; shaded areas indicate 95% confidence intervals of the regression fits.

**Figure 4:**
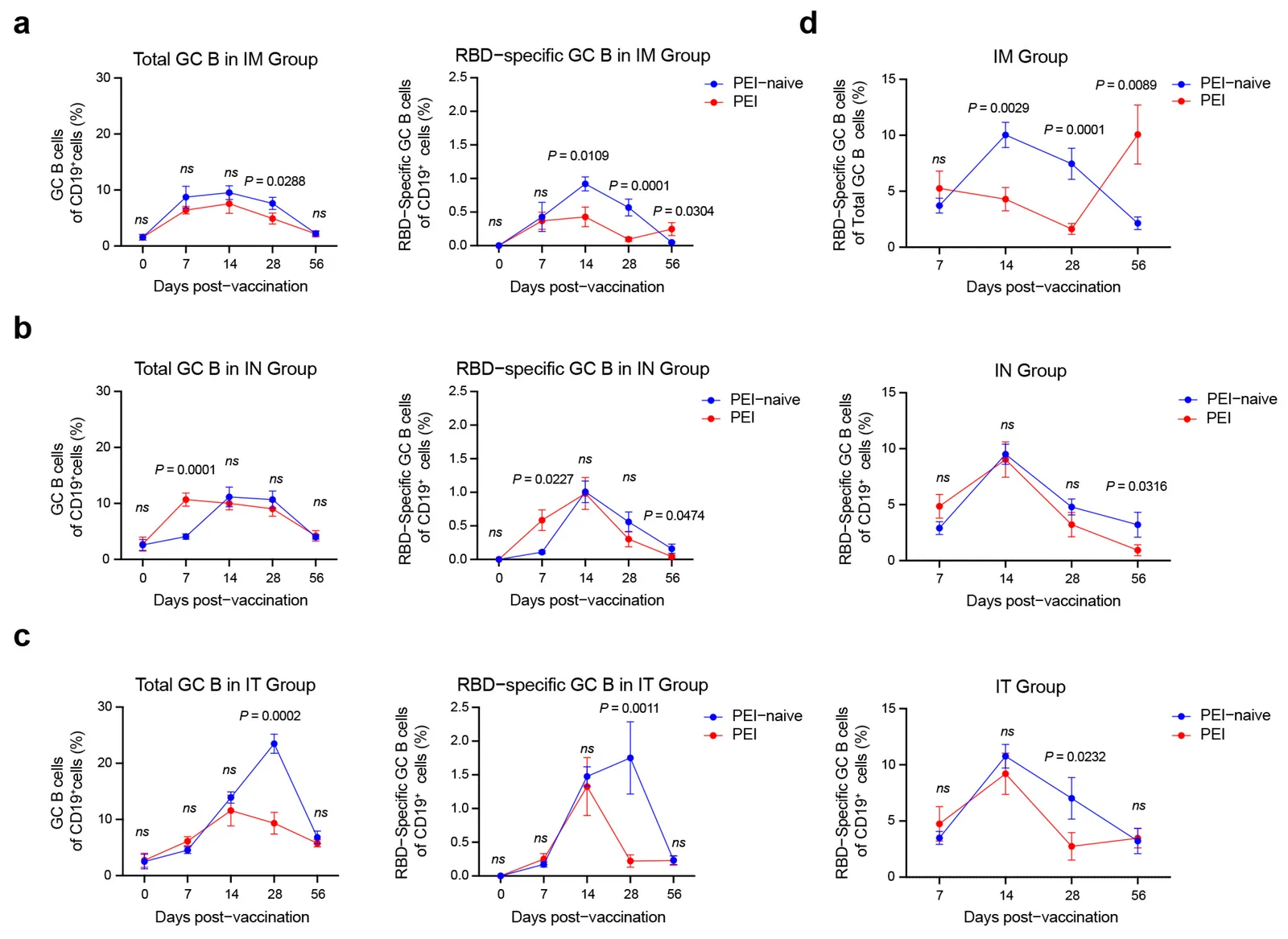
Germinal center B cell responses in draining lymph nodes after Ad5-nCoV immunization by three routes in PEI and PEI-naïve mice: (**a**–**c**) Frequencies of total GC B cells and RBD-specific GC B cells in the designated major lymph nodes of PEI (red) and PEI-naïve (blue) mice on days 0, 7, 14, 28, and 56 after immunization by the IM (**a**), IN (**b**), or IT (**c**) route. Points indicate group means, and error bars indicate the SEM. (**d**) Frequency of RBD-specific GC B cells among total GC B cells in PEI (red) and PEI-naïve (blue) mice on days 7, 14, 28, and 56 after vaccination by the IM, IN, or IT route. Data are presented as in (**a**–**c**). In (**a**–**d**), comparisons between PEI and PEI-naïve groups at each time point were performed using the unpaired Mann–Whitney U test. Exact *p* values are shown above the corresponding comparisons; ns indicates *p* ≥ 0.05.

**Figure 5:**
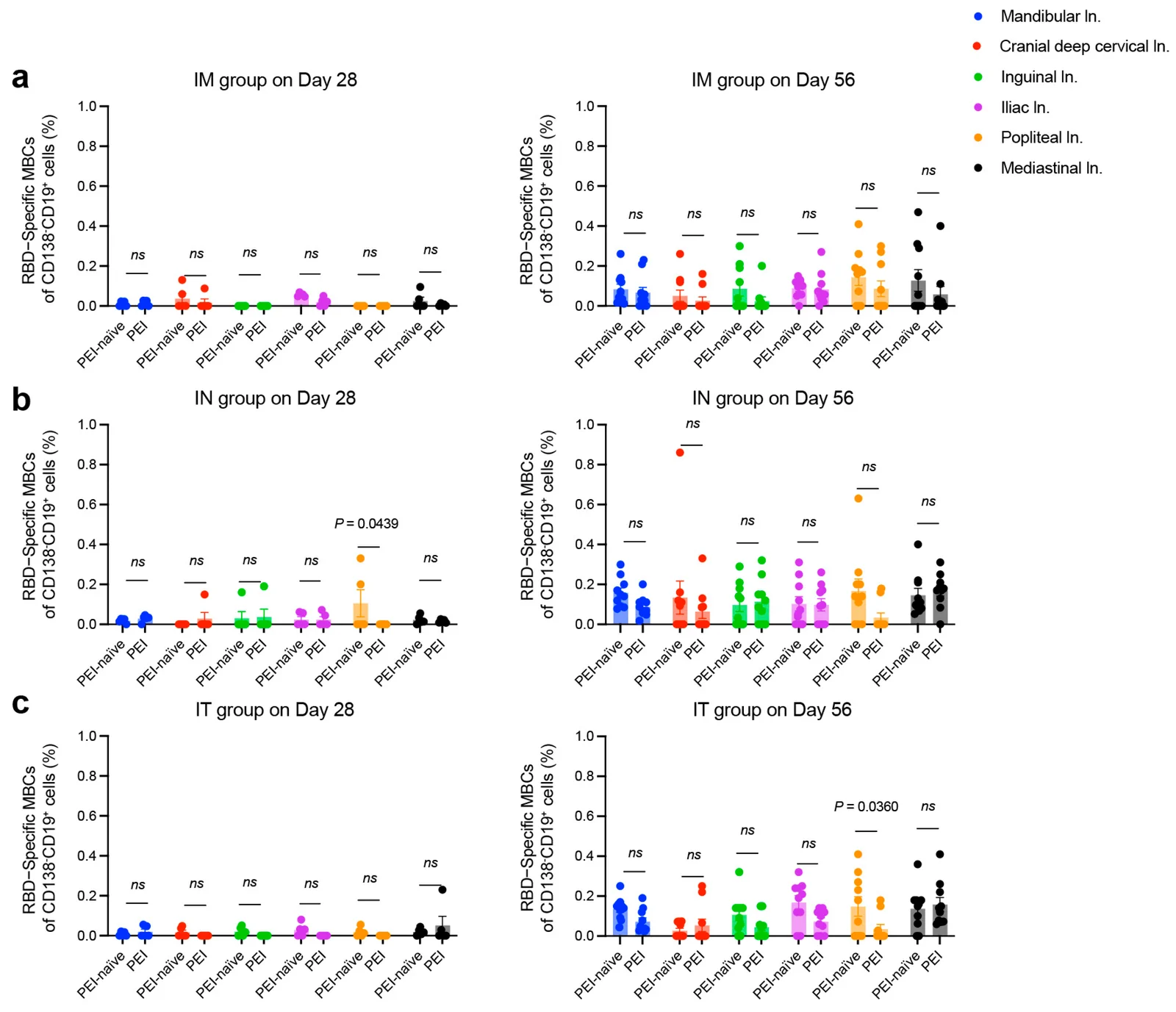
RBD-specific memory B cell responses in lymph nodes after Ad5-nCoV immunization by three routes in PEI and PEI-naïve mice: (**a**–**c**) Frequencies of RBD-specific MBCs in six lymph node stations in PEI and PEI-naïve mice on days 28 and 56 after immunization by the IM (**a**), IN (**b**), or IT (**c**) route. The lymph nodes examined were the mandibular, cranial deep cervical, inguinal, iliac, popliteal, and mediastinal lymph nodes. For each lymph node station, data are presented for four conditions: day 28 PEI-naïve, day 28 PEI, day 56 PEI-naïve, and day 56 PEI. Colors indicate the lymph node station. Bars indicate mean frequencies with SEM, and individual data points represent individual mice. Within each immunization route and each time point, two-way ANOVA followed by Sidak’s multiple-comparison test was performed to analyze the lymph node station and PEI status. Exact *p* values for multiple comparisons are shown above the corresponding comparisons; ns indicates *p* ≥ 0.05.

## Data Availability

The original contributions presented in the study are included in the article. Further inquiries can be directed to the corresponding authors.
